# Neurological Anatomy Applied to the Deltopectoral Surgical Approach: Safety Parameters in the Latarjet Procedure

**DOI:** 10.1055/s-0044-1800921

**Published:** 2025-03-12

**Authors:** André Leonardo Nogueira Farias, Leonardo Yabu Tanaka, Larissa Vasconcelos de Castro, Miguel Pereira da Costa, Romulo Brasil Filho, Antonio Carlos Tenor Júnior

**Affiliations:** 1 Grupo de Ombro e Cotovelo, Serviço de Ortopedia e Traumatologia, Hospital do Servidor Público Estadual (HSPE), São Paulo, SP, Brasil

**Keywords:** scapula/anatomy & histology, scapula/surgery, shoulder

## Abstract

**Objective** The present study aims to identify neurological safety parameters for performing the Latarjet procedure via the deltopectoral approach in a cross-sectional and prospective analysis of fresh cadavers.

**Methods** We dissected 12 shoulders from cadavers in good condition with no history of previous surgery or musculoskeletal dysfunction. Their mean age, height, weight, and body mass index (BMI) were the following: 75.33 (41–97) years, 168.81 (149–186) cm, 60.35 (26–77) kg, and 21.38 (11.71–34.22) kg/m ^2^ , respectively. We identified the anatomical landmark of the deltopectoral approach (medial glenoid rim, MGR) and measured its distance from the axillary, musculocutaneous, and subscapular nerves.

**Results** We obtained the following measurements in neutral rotation and 40° external rotation, respectively: distance from the MGR to the axillary nerve (AN), 2.87 cm and 2.58 cm ( *p* = 0.29); distance from the MGR to the musculocutaneous nerve (MCN), 2.70 cm and 2.54 cm ( *p* = 0.36); distance from the MGR to the upper subscapular nerve (USSN), 3.83 cm and 4.00 cm ( *p* = 0.30); distance from the MGR to the middle subscapular nerve (MSSN), 3.50 cm and 3.50 cm ( *p* = 1.00); and distance from the MGR to the lower subscapular nerve (LSSN), 3.00 cm and 2.83 cm ( *p* = 0.36).

**Conclusion** The deltopectoral approach is safe. However, in the Latarjet procedure, subscapularis muscle splitting and coracoid graft fixation require attention and caution due to the small distance to the adjacent nerves. These precautions can avoid major postoperative complications.

## Introduction

Latarjet surgery is a complex orthopedic procedure frequently used to treat recurrent shoulder instability. This procedure involves transferring the coracoid process to the glenoid cavity to stabilize the shoulder joint. Although effective in reducing instability, Latarjet surgery has risks and complications, including infection, nerve injury, joint stiffness, and graft nonunion. A careful approach and meticulous patient selection are essential to minimize these risks. 1

Neurological injuries are a significant concern in Latarjet surgery, and studies indicate a variable incidence of these complications. The literature highlights that neurological injuries potentially result from several factors, such as direct trauma during the surgical procedure, nerve compression due to an altered anatomical position after coracoid process transfer, and iatrogenic damage during graft fixation. These complications can lead to symptoms such as persistent pain, muscle weakness, and sensory deficits, significantly impacting the postoperative quality of life. Therefore, a careful approach, including identification and preservation of periarticular nerves, is critical to reduce the risk of neurologic injury during Latarjet surgery. 2 3

Yung et al. 4 specifically dissected the upper and lower subscapular nerves to study their role in innervating the subscapularis muscle. They described a safe zone for surgical dissection. These authors found that the palpable glenoid anterior rim, deep to the subscapularis, and the medial margin of the conjoint tendon could serve as landmarks because all neural branches were at least 1.5 cm medial to the conjoint tendon; in addition, all nerve branches to the subscapularis were on the anterior aspect of the muscular surface. 5 6 7

The muscular attachment site of the lower subscapular nerve is close to the axillary nerve, and its branches are small. Thus, the location and protection of the axillary nerve could guide the attachment point of the lower subscapular nerve. Interestingly, the lower subscapular nerve has recently been reported to arise directly from the axillary nerve segment in 21% of cases. 5 6 7

The Latarjet procedure, widely used for recurrent anterior glenohumeral joint dislocation, places the subscapular nerve at risk due to the sharp dissection of the subscapularis muscle for coracoid graft positioning, which can decrease adduction and the medial rotation strength of the arm. 8 The procedure also places the musculocutaneous nerve at risk during coracoid graft fixation with screws, potentially resulting in severe limitations to shoulder function. 9 10 11

The primary objective of the present study was to determine a “safety zone” for the subscapular nerve by measuring its distance from the coracoid graft using the medial glenoid rim (MGR) as a parameter. The secondary objective was to establish a “safety zone” for the axillary and musculocutaneous nerves by measuring their distance from the coracoid graft using MGR as a parameter.

## Materials and Methods

We submitted the project to the Ethics Committee of our institution in September 2023 and received its approval under CAAE No. 74541723.3.0000.5463.

**Fig. 1:**
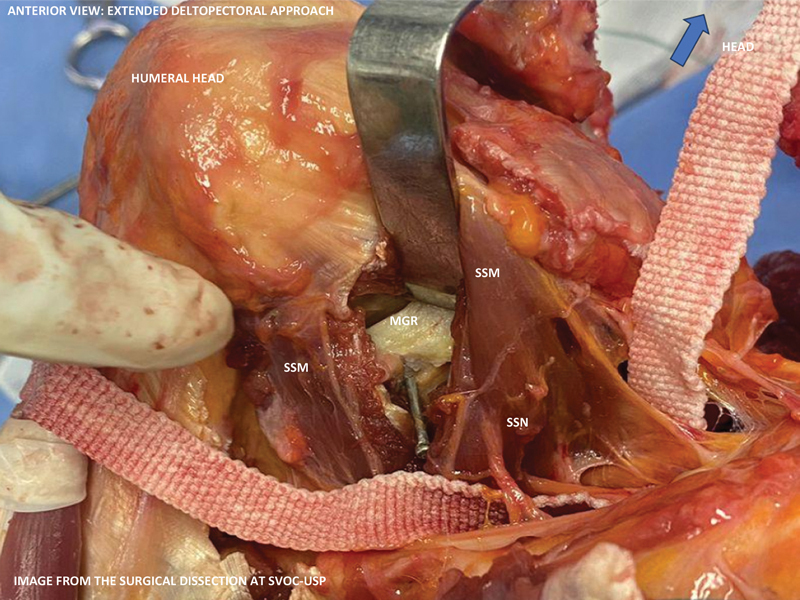
Surgical dissection performed at the São Paulo State Capital Death Verification Service (Serviço de Verificação de Óbitos da Capital, in Portuguese) from Universidade de São Paulo (SVOC-USP) in 2023: extended deltopectoral approach. **Abbreviations:** MGR, medial glenoid rim (marked with a metal pin); SSM, subscapularis muscle; SSN, subscapular nerves (marked with a cotton suture thread).

From October 2023 to January 2024, we dissected 12 shoulders (with no previous scars) from fresh adult cadavers. For dissections, we positioned the cadavers in dorsal decubitus with the shoulder in neutral rotation, the elbow flexed at 90°, and the forearm supinated at 45°. The dissection occurred through the deltopectoral groove. We performed juxtaosseous detachment and repair with high-resistance suture (Ethibond - Ethicon Inc., Raritan, NJ, USA) of the tendon of the conjoint tendon of the coracoid process. We divided the subscapularis muscle at the transition between the upper two thirds and the lower third of its muscular portion. Next, we fixated a plug measuring approximately 2.0 cm vertically at the MGR with two nails simulating the coracoid graft and fixation screws. We exposed the subscapularis muscle in its original anatomical position to measure three points on the internal surface of the muscle: the distance from the MGR to the course of the subscapular nerve (including the upper, middle, and lower branches); the distance from the MGR (at 3 o'clock considering the right shoulder) to the course of the axillary nerve; and the distance from the nails to the musculocutaneous nerve. All these measurements used a manual caliper graduated in millimeters ( Figs. 1 2 3 ). To measure the distance of the musculocutaneous nerve, we sutured the previously repaired conjoint tendon to the plug on the anterior glenoid rim, simulating the coracoid graft and maintaining the anatomical correlations of the surgery.

**Fig. 2:**
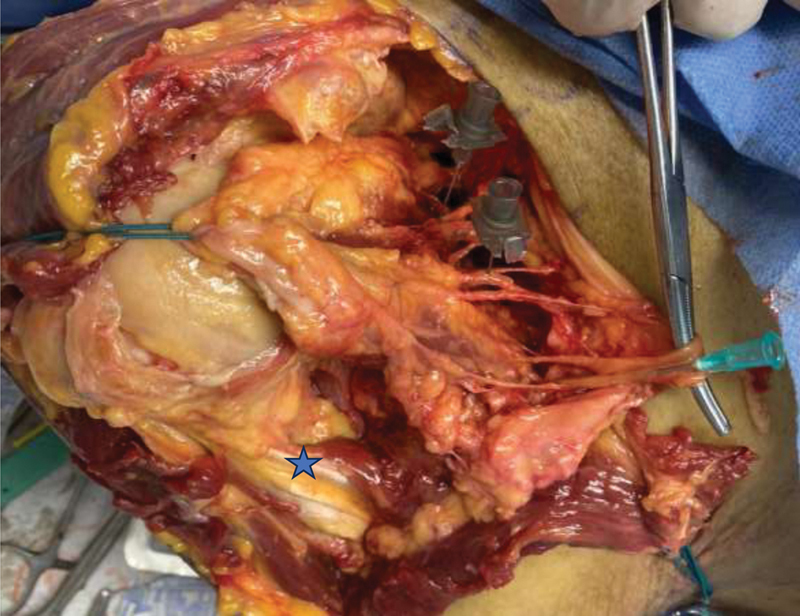
Surgical dissection performed at the SVOC-USP in 2023: extended deltopectoral approach. **Caption:** Subscapularis muscle detached and repaired with high-resistance suture; a gray needle marks the upper and middle subscapular nerves, and a green needle indicates the lower subscapular nerve; **Notes:** *Axillary nerve; lower/right region: conjoined tendon repaired with a high-resistance suture.

**Fig. 3:**
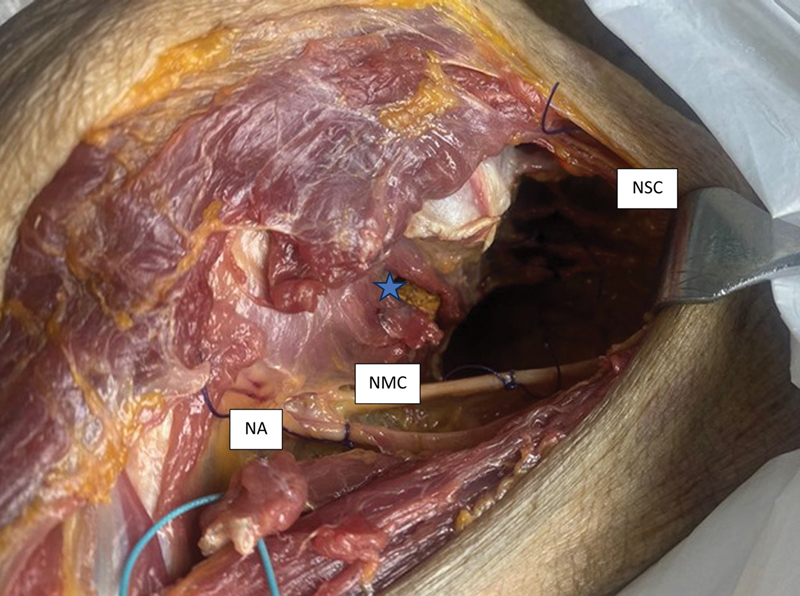
Surgical dissection performed at the SVOC-USP in 2023: extended deltopectoral approach. **Abbreviations:** SCN, subscapular nerves; MCN, musculocutaneous nerve, AN, axillary nerve; **Notes:** *Subscapular muscle split with graft); lower region: conjoint tendon repaired with a high-resistance suture.

All measurements occurred with the arm in a neutral position, as this is the most frequent position for shoulder surgery using the anterior or anterolateral approach, and in 40° external rotation, the average range of motion achieved by the limb after the Latarjet procedure.

### Sample description

We dissected 12 shoulders, with no previous scars, from 12 fresh adult cadavers from the Death Verification Service in our city. The cadavers included 4 females and 8 males, with a mean age of 75 years at the year of their death (95% confidence interval [95% CI]: 65–85), mean height of 168.9 cm (95% CI: 160–177), mean weight of 60.35 kg (95% CI: 48.9–71.7) and mean body mass index (BMI) of 21.38 (95% CI: 16.58–26.18) ( Table 1 ).

**Table 1:** Descriptive measures of the study sample

**a) Measurement summary (total sample)**			
**Variable**	**Mean**	**Standard deviation**	**95% confidence interval**
**Age (years)**	75.3	±15.7	65.3–85.3
**Height (cm)**	168.0	±12.8	160.0–177.0
**Weight (kg)**	60.3	±17.9	48.9–71.7
** Body mass index (kg/m ^2^ ) **	21.3	±7.5	16.58–26.18
**b) Measurement summary (female sex)**			
	**n**	**Mean(±standard deviation)**	**Minimum–maximum**
**Age (years)**	4	82.25 (±9.84)	77–97
**Height (cm)**	4	154.0(±8.67)	149–167
**Weight (kg)**	4	60(±24.04)	26–77
** Body mass index (kg/m ^2^ ) **	4	25.41(±10.92)	11.71–34.22
**c) Measurement summary (male sex)**			
	**n**	**Mean(±standard deviation)**	**Minimum-maximum**
**Age (years)**	8	71.88(±17.57)	41–95
**Height (cm)**	8	176.38(±6.19)	170–186
**Weight (kg)**	8	60.53(±16.15)	35.4–75
** Body mass index (kg/m ^2^ ) **	8	19.36(±4.95)	12.25–23.78
**d) Side frequency**			
**Side**	**n (%)**		
**Right**	7 (58.3%)		
**Left**	5 (41.6%)		
**e) Gender frequency**			
**Gender**	**n (%)**		
**Female**	4 (33.3%)		
**Male**	8 (66.6%)		

## Results

We dissected 12 shoulders, including 7 (53.3%) right shoulders and 5 (41.6%) left shoulders to determine the neurological anatomical measurements. These measurements occurred in neutral rotation and 40° external rotation of the shoulder, using the MGR as a landmark to evaluate, in millimeters, the distances between the MGR and the axillary nerve (AN), the musculocutaneous nerve (MCN), and the upper (USSN), middle (MSSN), and lower subscapular nerves (LSSN). Tables 2 and 3 show the mean measurements and the confidence intervals between the distances at the right and left shoulders in neutral and 40° rotation.

**Table 2:** Mean distances between the medial glenoid rim and neurological structures in the right and left shoulders in neutral rotation and 40° of lateral rotation

**a) Assessment in neutral rotation**						
	**Right shoulder**			**Left shoulder**		
	**Mean**	**Standard deviation**	**95% confidence interval**	**Mean**	**Standard deviation**	**95% confidence interval**
**Neutral rotation**						
**MCN**	2.57	±1.27	1.39–3.74	2.90	±1.24	1.35–4.45
**AN**	2.71	±0.48	1.29–3.41	3.10	±0.65	2.29–3.91
**USSN**	4.07	±1.23	2.92–5.21	3.50	±0.86	2.42–4.58
**MSSN**	3.78	±1.28	2.59–4.97	3.10	±0.41	2.58–3.62
**LSSN**	3.28	±0.99	2.36–4.20	2.60	±0.41	2.08–3.12
**b) Assessment in 40° lateral rotation**						
	**Right shoulder**			**Left shoulder**		
	**Mean**	**Standard deviation**	**95% confidence interval**	**Mean**	**Standard deviation**	**95% confidence interval**
**40° of rotation**						
**MCN**	2.42	±0.67	1.80–3.05	2.70	±1.44	0.91–4.49
**AN**	2.42	±1.20	1.31–3.54	2.80	±1.15	1.37–4.23
**USSN**	4.42	±1.01	3.48–5.36	3.40	±0.96	2.21–4.59
**MSSN**	3.71	±1.34	2.46–4.96	3.20	0.90	2.07–4.33
**LSSN**	3.28	±1.07	2.29–4.27	2.20	±0.57	1.49–2.91

Abbreviations: AN, axillary nerve; LSSN, lower suprascapular nerve in neutral rotation; MCN, musculocutaneous nerve; MSSN, middle suprascapular nerve; USSN, upper suprascapular nerve.

**Table 3:** Mean differences for left and right shoulders in neutral and 40° lateral rotation

	Right shoulder		Left shoulder		
	Mean	Standard deviation	Mean	Standard deviation	*p* -value
**MCN1**	2.57	± 1.2724	2.900	± 1.245	0.665926
**AN1**	2.714	± 0.488	3.100	± 0.6519	0.266182
**USSN1**	4.071	± 1.2392	3.500	± 0.866	0.397955
**MSSN1**	3.78	± 1.28	3.10	± 0.41	0.282479
**LSSN1**	3.28	± 0.99	2.60	± 0.41	0.180875
**MCN2**	2.42	± 0.67	2.70	± 1.44	0.668109
**AN2**	2.42	± 1.20	2.80	± 1.15	0.603784
**USSN2**	4.42	± 1.01	3.40	± 0.96	0.108165
**MSSN2**	3.71	± 1.34	3.20	± 0.90	0.478452
**LSSN2**	3.28	± 1.07	2.20	± 0.57	0.068181

**Abbreviations:** AN1, axillary nerve in neutral rotation; AN2, axillary nerve in 40° rotation; LSSN1, lower suprascapular nerve in neutral rotation; LSSN2, lower suprascapular nerve in 40° rotation; MCN1, musculocutaneous nerve in neutral rotation; MCN2, musculocutaneous nerve in 40° rotation; MSSN1, middle suprascapular nerve in neutral rotation; MSSN2, middle suprascapular nerve in 40° rotation; USSN1, upper suprascapular nerve in neutral rotation; USSN2, upper suprascapular nerve in 40° rotation.

The Student's t-test for mean values verified whether there was no difference between the anatomical measurements, regarding side, rotation, and sex. Before performing this test, we confirmed the assumption of normality using the Shapiro-Wilk test ( *p* -value > 0.05), which showed the measurement variables had a normal distribution between the right and left shoulders.

We detected no statistically significant differences ( *p* -value > 0.05) between the left and right shoulders regarding the distances from MGR and neurological structures in neutral or 40° rotation.

As for rotation, there were no statistically significant differences in the mean distances between shoulders in neutral and 40° rotation ( Tables 3 and 4 ). Regarding sex, we found no statistically significant differences ( Table 5 ).

**Table 4:** Mean differences in neutral and 40° lateral rotation

Variable	Mean	Standard deviation	N	Difference	t	*p*	95% CI	
**MCN1**	2.71	± 1.21						
**MCN2**	2.54	± 1.01	12	0.17	0.93808	0.368343	−0.22–0.56	
**AN1**	2.67	± 1.01						
**AN2**	2.58	± 1.14	12	0.08	0.48378	0.638029	−0.30–0.46	
**USSN1**	3.83	± 1.09						
**USSN2**	4.00	± 1.09	12	−0.17	−1.07606	0.304937	−0.51–0.17	
**MSSN1**	3.50	± 1.04						
**MSSN2**	3.50	± 1.17	12	−0.00	−0.00000	1.000000	−0.43–0.43	
**LSSN1**	3.00	± 0.85						
**LSSN2**	2.83	± 1.03	12	0.17	0.93808	0.368343	−0.22–0.56	

**Abbreviations:** 95% CI, 95% confidence interval; AN1, axillary nerve in neutral rotation; AN2, axillary nerve in 40° rotation; LSSN1, lower suprascapular nerve in neutral rotation; LSSN2, lower suprascapular nerve in 40° rotation; MCN1, musculocutaneous nerve in neutral rotation; MCN2, musculocutaneous nerve in 40° rotation; MSSN1, middle suprascapular nerve in neutral rotation; MSSN2, middle suprascapular nerve in 40° rotation; USSN1, upper suprascapular nerve in neutral rotation; USSN2, upper suprascapular nerve in 40° rotation.

**Table 5:** Measurement difference per sex

	Female sex		Male sex		
	Mean	Standard deviation	Mean	Standard deviation	*p* -value
**MCN1**	2.625	± 1.1087	2.750	± 1.3363	0.875731
**AN1**	2.75	± 0.8660	2.938	± 0.4173	0.614463
**USSN1**	3.875	± 0.2500	3.813	± 1.3611	0.930854
**MSSN1**	3.125	± 0.2500	3.688	± 1.2518	0.404879
**LSSN1**	2.625	± 0.4787	3.188	± 0.9613	0.303033
**MCN2**	2.75	± 0.9574	2.438	± 1.0836	0.636582
**AN2**	2.875	± 1.2500	2.438	± 1.1476	0.558121
**USSN2**	4.000	± 0.4082	4.000	± 1.3363	1.00000
**MSSN2**	2.875	± 1.0308	3.813	± 1.1630	0.203447
**LSSN2**	2.125	± 0.6292	3.188	± 1.0329	0.091778

**Abbreviations:** AN1, axillary nerve in neutral rotation; AN2, axillary nerve in 40° rotation; LSSN1, lower suprascapular nerve in neutral rotation; LSSN2, lower suprascapular nerve in 40° rotation; MCN1, musculocutaneous nerve in neutral rotation; MCN2, musculocutaneous nerve in 40° rotation; MSSN1, middle suprascapular nerve in neutral rotation; MSSN2, middle suprascapular nerve in 40° rotation; USSN1, upper suprascapular nerve in neutral rotation; USSN2, upper suprascapular nerve in 40° rotation.

## Discussion

Neurological injuries during Latarjet surgery are concerning, and several sources from the literature highlight the significance of anatomical and dissection aspects to prevent them. Authors such as Lafosse and Boyle 12 and Nam et al. 3 emphasize the need to fully understand the anatomy of periarticular nerves, including the suprascapular and the axillary nerves, to minimize the risk of injuries during the surgical procedure. Meticulous dissection techniques, as described by Maldonado et al., 13 are crucial to preserve nerve integrity during coracoid process transfers. In addition, strategies such as nerve identification and adequate protection during graft fixation are recommended, as discussed by Gupta et al., 14 Domos et al., 15 Ferreira Filho et al., 16 and da Silva et al. 17 These combined approaches help to effectively reduce the risk of neurological injuries and improve postsurgical outcomes.

The present study is consistent with the one by Coifman et al., 18 who reported mean distances between the MGR and the upper, middle, and lower portions of the subscapular nerve in neutral rotation as 33 mm, 32.5 mm, and 20 mm, respectively. This study also agrees with that of La Prade et al., 19 who described that the mean distances between the MGR and the musculocutaneous and axillary nerves were 24.4 mm and 19.8 mm, respectively. Most measurements were within the confidence interval of this study, except for the lower portion of the subscapular nerve reported by Coifman et al. 18 This difference may result from the fact that these authors made their measurements on formalized pieces, potentially reducing the course of the structures.

In contrast, the studies from other authors used smaller samples with different epidemiological characteristics. La Prade et al. 19 analyzed 14 shoulders of 7 North American cadavers, all males, with an average age of 53. Coifman et al. 18 analyzed 12 shoulders of 6 Hispanic cadavers, with an average age of 74 years, including 4 females and 2 males. Our study brought innovations regarding rotational measurements and comparisons for side and sex, aspects disregarded by previous authors.

The neurological complication rate after the Latarjet procedure ranges from 8 to 10% in clinical studies; these complications mostly affect the axillary and musculocutaneous nerves. 2 9 20 It is believed that neurological injury after the bone block procedure results from traction, poor patient positioning, and inadvertent suturing. Most injuries were transient neuropraxia, with no long-term clinical repercussions. Furthermore, the authors believe that the neurological complication rate may be higher because of the lack of adequate recording. 2 20

In the Latarjet procedure, in addition to filling the glenoid bone defect with the coracoid graft, the “sling” effect of the conjoint tendon transferred between a split in the subscapularis muscle is a critical “soft-tissue stabilizer” for the humeral head. However, it has been shown that the lower part of the subscapularis muscle tends to degenerate, leading to fatty infiltration of the muscle. In addition, exposure through subscapularis splitting is technically demanding and implies a risk of nerve damage due to the traction forces of the retractors during open surgery. 21

The long-term structural and functional integrity of the subscapularis after the Latarjet procedure remains unknown. 22 23 In a mean follow-up period of 8 years after the Latarjet procedure, Azizi et al. 22 found a slight reduction in abduction strength, but no reduction in medial rotation strength. Furthermore, in ultrasound and magnetic resonance imaging studies, the subscapularis tendon remained intact in circumference compared with preoperative measurements. In contrast, Ernstbrunner et al. 23 found a significant decrease in active medial and lateral rotation and strength compared with the healthy contralateral shoulder. However, the clinical influence of these findings has not yet been defined. There was no increase in fatty degeneration of the subscapularis muscle but minimal hypertrophy on the operated side in the long-term follow-up.

The discussion about the integrity of the subscapularis muscle and its innervation in the Latarjet procedure is growing in the literature. Davey et al. 24 analyzed, in a systematic review, whether managing the subscapularis muscle with a split or “L” tenotomy would influence the clinical outcomes of the open Latarjet procedure through a deltopectoral approach. This systematic review established that the subscapularis splitting technique resulted in significantly better functional outcome measures and lower rates of subscapularis insufficiency compared with an “L” tenotomy technique in the medium-term follow-up. 24 Furthermore, Raiss et al. 21 described the procedure known as “Flipped Latarjet”, that is, the transfer of the coracoid process to the deficient glenoid with no subscapularis muscle splitting, maintaining the benefits of a sling effect of the conjoint tendon. Although there are no long-term data available to prove any superiority of the method compared with the traditional open or arthroscopic Latarjet procedure, this technique would be an alternative to avoid neuromuscular injuries to the subscapularis.

The limitations of this study are sample size due to the inherent difficulty of cadaveric studies, anatomical variations in the brachial plexus, and loss of tension in neurological structures since we used fresh cadavers with no fixation techniques for the shoulder girdle and humerus. Furthermore, the average age of the cadavers is far from the target age for the Latarjet technique, potentially modifying muscle quality and volume.

Finally, this is a pilot study whose main purpose was to verify average neurological distances, and the same observer performed all measurements more than once to ensure data reliability. A supplementary analysis is under development, with a larger sample size and another design type to assess the interobserver measurement reliability using the Kappa coefficient.

## Conclusion

The deltopectoral approach is safe. However, in the Latarjet procedure, subscapularis muscle splitting, and coracoid graft fixation require attention and caution due to the small distance to the adjacent nerves. These precautions can avoid major postoperative complications.
