# Supplementary material for: Neurological Anatomy Applied to the Deltopectoral Surgical Approach: Safety Parameters in the Latarjet Procedure
Source: Rev Bras Ortop (Sao Paulo). 2025 Mar 12;60(1):s00441800921. doi: 10.1055/s-0044-1800921 (PMC11903118; doi:10.1055/s-0044-1800921)
Supplement: Related texts

# Research article: Anatomia neurológica aplicada à abordagem cirúrgica deltopeitoral: Parâmetros de segurança no procedimento de Latarjet

**Keywords:** escápula/anatomia & histologia, escápula/cirurgia, ombro

## Abstract

**Objetivo** Identificar parâmetros de segurança neurológica para a realização do procedimento de Latarjet através da via deltopeitoral por meio de um estudo transversal e prospectivo em cadáveres frescos.

**Métodos** Foram dissecados 12 ombros de cadáveres, sem história de cirurgia ou disfunção musculoesquelética prévia e em bom estado de conservação, com médias de idade, altura, peso e índice de massa corporal (IMC): 75,33 (41–97) anos; 168,81 (149–186) cm; 60,35 (26–77) kg e 21,38 (11,71–34,22) kg/m ^2^ , respectivamente. Foi identificado o marco anatômico da via estudada (rebordo medial da glenoide - RMG) e sua distância para os nervos axilar, musculocutâneo e subescapular foi mensurada.

**Resultados** Obtivemos as seguintes medidas em rotação neutra e rotação externa de 40°, respectivamente: a distância entre o RMG e o nervo axilar (NA) = 2,87 cm e 2,58 cm ( *p* = 0,29); a distância do RMG ao nervo musculocutâneo (NMC) = 2,70 cm e 2,54 cm ( *p* = 0,36); a distância do RMG ao nervo subescapular superior (NSS) = 3,83 cm e 4,00 cm ( *p* = 0,30); a distância do RMG ao nervo subescapular médio (NSM) = 3,50 cm e 3,50 cm ( *p* = 1,00); e a distância do RMG ao nervo subescapular inferior (NSI) = 3,00 e 2,83 cm ( *p* = 0,36).

**Conclusão** A via deltopeitoral é considerada segura; porém, é preciso atenção e cautela durante o *split* do músculo subescapular e na fixação do enxerto do coracoide no procedimento de Latarjet, devido à curta distância até os nervos adjacentes. Tais precauções podem evitar maiores complicações pós-operatórias.

## Introdução

A cirurgia de Latarjet é um procedimento ortopédico complexo frequentemente utilizado no tratamento de instabilidade recorrente do ombro. Este procedimento envolve a transferência do processo coracoide para a cavidade glenoidal, visando estabilizar a articulação do ombro. Embora seja eficaz na redução da instabilidade, a cirurgia de Latarjet não está isenta de riscos e complicações. Estes incluem infecção, lesão nervosa, rigidez articular e não união do enxerto. Uma abordagem cuidadosa e uma seleção criteriosa de pacientes são essenciais para minimizar esses riscos. 1

As lesões neurológicas são uma preocupação significativa associada à cirurgia de Latarjet, com estudos indicando uma incidência variável dessas complicações. A literatura destaca que as lesões neurológicas podem resultar de diversos fatores, como trauma direto durante o procedimento cirúrgico, compressão nervosa devido à posição anatômica alterada após a transferência do processo coracoide e iatrogenia durante a fixação do enxerto. Essas complicações podem levar a sintomas como dor persistente, fraqueza muscular e déficits sensoriais, impactando significativamente na qualidade de vida pós-operatória do paciente. Portanto, uma abordagem cuidadosa, incluindo a identificação e preservação dos nervos periarticulares, é fundamental para reduzir o risco de lesões neurológicas durante a cirurgia de Latarjet. 2 3

Yung et al. 4 dissecaram especificamente os nervos subescapulares superior e inferior em relação à sua inervação do músculo subescapular. Eles descreveram uma zona segura para dissecção cirúrgica. Eles descobriram que o rebordo anterior palpável da glenoide, profunda ao subescapular, juntamente com a margem medial do tendão conjunto poderiam servir como pontos de referência porque todos os ramos neurais foram pelo menos 1,5 cm mediais ao tendão conjunto e todos os ramos nervosos para o subescapular estavam na parte anterior da superfície muscular. 5 6 7

O local de inserção muscular do nervo subescapular inferior é próximo ao nervo axilar e seus ramos são muito pequenos. Deste modo, a localização e proteção do nervo axilar poderia servir como um guia para o ponto de inserção do nervo subescapular inferior. Curiosamente, foi recentemente relatado que o nervo subescapular inferior se origina diretamente do segmento do nervo axilar em 21% dos casos. 5 6 7

O procedimento de Latarjet, amplamente utilizado em casos de luxação anterior recidivante da articulação gleno-umeral, coloca o nervo subescapular em risco pela dissecção cortante do músculo subescapular para posicionamento do enxerto do coracoide, o que pode resultar na diminuição da força de adução e rotação medial do braço. 8 O procedimento põe em risco ainda o nervo musculocutâneo durante a fixação do enxerto do coracoide com parafusos, também podendo resultar em limitações severas para a função do ombro. 9 10 11

O objetivo primário deste trabalho é determinar uma “zona de segurança” para o nervo subescapular, através da medida da sua distância ao enxerto do coracoide, usando como parâmetro o rebordo medial da glenoide. São objetivos secundários: estabelecer uma “zona de segurança” para os nervos axilar e musculocutâneo, através da medida da sua distância ao enxerto do coracoide, usando como parâmetro o rebordo medial da glenoide.

## Materiais e Métodos

O projeto foi submetido ao Comitê de Ética da nossa instituição em setembro de 2023 e aprovado sob CAAE n° 74541723.3.0000.5463. No período de outubro de 2023 a janeiro de 2024, foram dissecados 12 ombros (sem história de cicatrizes prévias) de cadáveres adultos, frescos.

Durantes as dissecções, os cadáveres foram posicionados em decúbito dorsal com o ombro em rotação neutra, o cotovelo fletido a 90° e o antebraço supinado a 45°. A dissecção foi realizada através do sulco deltopeitoral. Foi realizada a desinserção justaossea e reparo com fio de alta resistência (Ethibond- Ethicon Inc., Raritan, NJ, EUA) do tendão conjunto do processo coracoide. Foi realizado o *split* do músculo subescapular na transição entre os 2/3 superiores e o 1/3 inferior de sua porção muscular. Em seguida, foi realizada a fixação de uma rolha de aproximadamente 2,0 cm em posição vertical no rebordo medial da glenoide com dois pregos simulando o enxerto do coracoide e os parafusos de fixação. O músculo subescapular foi exposto em sua posição anatômica original, e três pontos foram mensurados na superfície interna do músculo: A distância do rebordo medial da glenoide ao curso do nervo subescapular (incluindo os ramos superior/médio/ inferior), a distância do RMG (às 3h considerando o ombro direito) ao curso do nervo axilar e a distância dos pregos ao nervo musculocutâneo, com o uso de paquímetro manual graduado em milímetros ( Figs. 1 2 3 ). Para as mensurações da distância do nervo musculocutaneo, o tendão conjunto previamente reparado foi suturado na rolha situada no rebordo anterior da glenoide, simulando o enxerto do coracoide e mantendo as correlações anatômicas da cirurgia.

**Fig. 1:** Dissecção cirúrgica realizada no Serviço de Verificação de Óbitos da Capital da Universidade de São Paulo (SVOC-USP) em 2023: acesso deltopeitoral extendido. **Abreviações:** RMG, rebordo medial da glenoide (demarcado com pino metálico); M. SSC, músculo subescapular; NN. Subescapulares, nervos subescapulares (demarcados com fio de algodão).

**Fig. 2:** Dissecção cirúrgica realizada no SVOC-USP em 2023: acesso deltopeitoral extendido. **Legenda:** Músculo subescapular desinserido e reparado com fio de alta resistência; demarcado com agulha cinza nn. subescapulares superior e médio; demarcado com agulha verde n. subescapular inferior; **Notas:** *Nervo axilar; na região inferior/direita: tendão conjunto reparado com fio de alta resistência.

**Fig. 3:** Dissecção cirúrgica realizada no SVOC-USP em 2023: acesso deltopeitoral extendido. **Abreviações:** NSC, nervos subescapulares; NMC, nervo musculocutâneo, NA, nervo axilar; **Notas:** * *Split* do músculo subescapular com enxerto; na região inferior: tendão conjunto reparado com fio de alta resistência.

Todas a mensurações foram realizadas mantendo o braço em posição neutra, pois esta é a posição mais comumente usada para cirurgia do ombro usando abordagem anterior ou anterolateral e em rotação externa de 40°, que é o arco de movimento médio alcançado pelo membro após o procedimento de Latarjet.

## Descrição da amostra

Foram dissecados 12 ombros, sem história de cicatrizes prévias, de 12 cadáveres adultos, frescos, no Serviço de Verificação de Óbitos da nossa cidade. Os cadáveres incluíam 4 pacientes do sexo feminino e 8 pacientes do sexo masculino, com idade média de 75 anos no ano da morte (intervalo de confiança de 95% [IC 95%]: 65–85), altura média de 168,9 cm (IC 95%: 160–177), peso médio de 60,35 Kg (IC 95%: 48,9–71,7), e índice de massa corporal (IMC) médio de 21,38 (IC 95%: 16,58–26,18) ( Tabela 1 ).

**Tabela 1:** Medidas descritivas da amostra do estudo

**a) Medidas de resumo (amostra total)**			
**Variável**	**Média**	**Desvio padrão**	**Interalo de confiança de 95%**
**Idade (anos)**	75,3	±15,7	65,3–85,3
**Altura (cm)**	168,0	±12,8	160,0–177,0
**Peso (kg)**	60,3	±17,9	48,9–71,7
** Índice de massa corporal (kg/m ^2^ ) **	21,3	±7,5	16,58–26,18
**b) Medidas de resumo (sexo feminino)**			
	**n**	**Média(±desvio padrão)**	**Mínimo–máximo**
**Idade (anos)**	4	82,25(±9,84)	77–97
**Altura (cm)**	4	154,0(±8,67)	149–167
**Peso (kg)**	4	60(±24,04)	26–77
** Índice de massa corporal (kg/m ^2^ ) **	4	25,41(±10,92)	11,71–34,22
**c) Medidas de resumo (sexo masculino)**			
	**n**	**Média(±desvio padrão)**	**Mínimo–máximo**
**Idade (anos)**	8	71,88(±17,57)	41–95
**Altura (cm)**	8	176,38(±6,19)	170–186
**Peso (kg)**	8	60,53(±16,15)	35,4–75
** Índice de massa corporal (kg/m ^2^ ) **	8	19,36(±4,95)	12,25–23,78
**d) Frequência de lateralidade**			
**Lateralidade**	**n (%)**		
**Direita**	7 (58,3%)		
**Esquerda**	5 (41,6%)		
**e) Frequência de sexo**			
**Sexo**	**n (%)**		
**Feminino**	4 (33,3%)		
**Masculino**	8 (66,6%)		

## Resultados

Para o estudo de medidas anatômicas neurológicas, foram dissecados 12 ombros, sendo 7 do lado direito (53,3%) e 5 do lado esquerdo (41,6%). As medidas foram realizadas em rotação neutra e rotação externa de 40° do ombro, utilizando o RMG como ponto referencial para avaliar em milímetros as distâncias entre o RMG e o nervo axilar (NA), o nervo musculocutâneo (NMC), e os nervos subescapulares superior (SS), médio (SM) e inferior (SI), respectivamente. As Tabelas 2 e 3 descrevem os resultados das medidas de tendência e os intervalos de confiança entre as distâncias, para ombro direito e esquerdo em rotação neutra e rotação em 40°.

**Tabela 2:** Medidas de tendência para distâncias entre o rebordo medial da glenoide e estruturas neurológicas nos ombros direito e esquerdo para rotação neutra e rotação em rotação lateral de 40°

**a) Avaliação em rotação neutra**						
	**Ombro direito**			**Ombro esquerdo**		
	**Média**	**Desvio padrão**	**IC 95%**	**Média**	**Desvio padrão**	**IC 95%**
**Rotação neutra**						
**NMC**	2,57	±1,27	1,39–3,74	2,90	±1,24	1,35–4,45
**NA**	2,71	±0,48	1,29–3,41	3,10	±0,65	2,29–3,91
**SS**	4,07	±1,23	2,92–5,21	3,50	±0,86	2,42–4,58
**SM**	3,78	±1,28	2,59–4,97	3,10	±0,41	2,58–3,62
**SI**	3,28	±0,99	2,36–4,20	2,60	±0,41	2,08–3,12
**b) Avaliação em rotação lateral de 40°**						
	**Ombro direito**			**Ombro esquerdo**		
	**Média**	**Desvio padrão**	**IC 95%**	**Média**	**Desvio padrão**	**IC 95%**
**Rotação em 40°**						
**NMC**	2,42	±0,67	1,80–3,05	2,70	±1,44	0,91–4,49
**NA**	2,42	±1,20	1,31–3,54	2,80	±1,15	1,37–4,23
**SS**	4,42	±1,01	3,48–5,36	3,40	±0,96	2,21–4,59
**SM**	3,71	±1,34	2,46–4,96	3,20	±0,90	2,07–4,33
**SI**	3,28	±1,07	2,29–4,27	2,20	±0,57	1,49–2,91

Abreviações: IC 95%, intervalo de confiança de 95%; NA, nervo axiliar; NMC, nervo musculocutâneo; SS, supraescapular superior; SM, supraescapular médio, SI, supraescapular inferior.

**Tabela 3:** Diferença entre médias para ombros esquerdos e direito em rotação neutra e rotação lateral de 40°

	Ombro direito		Ombro esquerdo		
	Média	Desvio padrão	Média	Desvio padrão	Valor de *p*
**NMC1**	2,57	± 1,2724	2,900	± 1,245	0,665926
**NA1**	2,714	± 0,488	3,100	± 0,6519	0,266182
**SS1**	4,071	± 1,2392	3,500	± 0,866	0,397955
**SM1**	3,78	± 1,28	3,10	± 0,41	0,282479
**SI1**	3,28	± 0,99	2,60	± 0,41	0,180875
**NMC2**	2,42	± 0,67	2,70	± 1,44	0,668109
**NA2**	2,42	± 1,20	2,80	± 1,15	0,603784
**SS2**	4,42	± 1,01	3,40	± 0,96	0,108165
**SM2**	3,71	± 1,34	3,20	± 0,90	0,478452
**SI2**	3,28	± 1,07	2,20	± 0,57	0,068181

**Abreviações:** NA1, nervo axiliar em rotação neutra; NA2, nervo axiliar em rotação 40°; NMC1, nervo musculocutâneo em rotação neutra; NMC2, nervo musculocutâneo em rotação 40°; SI1, nervo supraescapular inferior em rotação neutra; SI2, nervo supraescapular inferior em rotação 40°; SM1, nervo supraescapular médio em rotação neutra; SM2, nervo supraescapular médio em rotação 40°; SS1, nervo supraescapular superior em rotação neutra; , SS2, nervo supraescapular superior em rotação 40°.

Para verificar se não há diferença entre as medidas anatômicas, quanto à lateralidade, à rotação e ao sexo, foi realizado o teste t de Student para médias. Antes da realização do teste, o pressuposto de normalidade foi confirmado pelo teste de Shapiro-Wilk (valor de *p* > 0,05), de acordo com o qual as variáveis de medida possuem distribuição normal entre ombro direito e esquerdo.

Quanto à lateralidade, não foram observadas diferenças estatisticamente significativas (valor de *p* > 0,05) entre os ombros esquerdo e direito, para as distâncias entre o RMG e estruturas neurológicas para rotação neutra e rotação em 40°.

Em relação à rotação, não foram encontradas diferenças estatisticamente significativas, entre as médias das distancias entre ombros em rotação neutra e rotação em 40° ( Tabelas 3 e 4 ). Em relação ao sexo, também não foram encontradas diferenças estatisticamente significativas ( Tabela 5 ).

**Tabela 4:** Diferença entre médias entre rotação neutra e rotação lateral de 40°

Variável	Média	Desvio padrão	N	Diferença	t	*p*	IC 95%	
**NMC1**	2,71	± 1,21						
**NMC2**	2,54	± 1,01	12	0,17	0,93808	0,368343	−0,22–0,56	
**NA1**	2,67	± 1,01						
**NA2**	2,58	± 1,14	12	0,08	0,48378	0,638029	−0,30–0,46	
**SS1**	3,83	± 1,09						
**SS2**	4,00	± 1,09	12	−0,17	−1,07606	0,304937	−0,51–0,17	
**SM1**	3,50	± 1,04						
**SM2**	3,50	± 1,17	12	−0,00	−0,00000	1,000000	−0,43–0,43	
**SI1**	3,00	± 0,85						
**SI2**	2,83	± 1,03	12	0,17	0,93808	0,368343	−0,22–0,56	

**Abreviações:** IC 95%, intervalo de confiança de 95%; NA1, nervo axiliar em rotação neutra; NA2, nervo axiliar em rotação 40°; NMC1, nervo musculocutâneo em rotação neutra; NMC2, nervo musculocutâneo em rotação 40°; SI1, nervo supraescapular inferior em rotação neutra; SI2, nervo supraescapular inferior em rotação 40°; SM1, nervo supraescapular médio em rotação neutra; SM2, nervo supraescapular médio em rotação 40°; SS1, nervo supraescapular superior em rotação neutra; , SS2, nervo supraescapular superior em rotação 40°.

**Tabela 5:** Diferença de medidas entre os sexos

	Sexo feminino		Sexo masculino		
	Média	Desvio padrão	Média	Desvio padrão	Valor de *p*
**NMC1**	2,625	± 1,1087	2,750	± 1,3363	0,875731
**NA1**	2,75	± 0,8660	2,938	± 0,4173	0,614463
**SS1**	3,875	± 0,2500	3,813	± 1,3611	0,930854
**SM1**	3,125	± 0,2500	3,688	± 1,2518	0,404879
**SI1**	2,625	± 0,4787	3,188	± 0,9613	0,303033
**NMC2**	2,75	± 0,9574	2,438	± 1,0836	0,636582
**NA2**	2,875	± 1,2500	2,438	± 1,1476	0,558121
**SS2**	4,000	± 0,4082	4,000	± 1,3363	1,00000
**SM2**	2,875	± 1,0308	3,813	± 1,1630	0,203447
**SI2**	2,125	± 0,6292	3,188	± 1,0329	0,091778

**Abreviações:** NA1, nervo axiliar em rotação neutra; NA2, nervo axiliar em rotação 40°; NMC1, nervo musculocutâneo em rotação neutra; NMC2, nervo musculocutâneo em rotação 40°; SI1, nervo supraescapular inferior em rotação neutra; SI2, nervo supraescapular inferior em rotação 40°; SM1, nervo supraescapular médio em rotação neutra; SM2, nervo supraescapular médio em rotação 40°; SS1, nervo supraescapular superior em rotação neutra; , SS2, nervo supraescapular superior em rotação 40°.

## Discussão

As lesões neurológicas durante a cirurgia de Latarjet são uma preocupação, ressaltando-se a importância dos aspectos anatômicos e de dissecação para preveni-las, conforme destacado em várias fontes da literatura. Estudos como o de Lafosse e Boyle 12 e Nam et al. 3 enfatizam a necessidade de compreender detalhadamente a anatomia dos nervos periarticulares, como o nervo supraescapular e o nervo axilar, para minimizar o risco de lesões durante o procedimento cirúrgico. Técnicas meticulosas de dissecação, conforme descritas por Maldonado et al., 13 são cruciais para preservar a integridade nervosa durante a transferência do processo coracoide. Além disso, estratégias como a identificação dos nervos e a proteção adequada durante a fixação do enxerto são recomendadas, como discutido nos estudos de Gupta et al., 14 Domos et al., 15 Ferreira Filho et al. 16 e da Silva et al. 17 Essas abordagens combinadas ajudam a reduzir efetivamente o risco de lesões neurológicas e melhoram os resultados pós-cirúrgicos.

Este estudo está em concordância com o trabalho realizado por Coifman et al. 18 com evidência da distância média entre o RMG e as porções dos nervos subescapular em rotação neutra, isto é, superior = 33 mm, média = 32.5 mm e inferior 20 mm. Ademais, este estudo está em concordância com o trabalho feito por La Prade et al. 19 com evidência da distância média entre o RMG o nervo musculocutâneo = 24.4 mm e o nervo axilar = 19.8 mm. A maioria das medidas encontrou-se dentro do IC deste estudo, exceto a porção inferior do nervo subescapular relatada por Coifman et al. 18 Essa diferença de resultados poderia ser justificada pelo fato de esses autores terem feito suas aferições em peças formolizadas, o que possivelmente reduziu a distância entre as estruturas.

Por outro lado, os trabalhos desenvolvidos por outros autores foram realizados com amostras menores e com características epidemiológicas diferentes. La Prade et al. 19 analisaram 14 ombros de 7 cadáveres norte-americanos, com idade média de 53 anos e apenas do sexo masculino. Coifman et al. 18 analisaram 12 ombros de 6 cadáveres hispânicos, com idade média de 74 anos, sendo 4 mulheres e 2 homens. Este estudo trouxe de pontos inovadores a aferição rotacional e comparação quanto lateralidade/sexo da amostra, aspectos desconsiderados pelos autores anteriores.

As complicações neurológicas após o procedimento de Latarjet variam entre 8 e 10% nos estudos clínicos, sendo os nervos axilar e musculocutaneo os mais acometidos. 2 9 20 Acredita-se que a lesão neurológica após o procedimento de bloqueio ósseo seja ocasionada por tração, mau posicionamento do paciente e sutura inadvertida. A maioria das lesões foram neuropraxias transitórias, sem repercussões clínicas a longo prazo. Ademais, os autores acreditam que as taxas de complicações neurológicas podem ser ainda maiores, pois na maioria dos casos não haveria registro adequado desses casos. 2 20

No procedimento de Latarjet, além do preenchimento do defeito ósseo glenoidal com o enxerto do coracoide, o efeito “sling” do tendão conjunto transferido entre uma divisão no músculo subescapular é um importante “estabilizador de tecidos moles” da cabeça do úmero. No entanto, foi demonstrado que a parte inferior do músculo subescapular tende a degenerar, levando à infiltração gordurosa do próprio músculo. Além disso, a exposição através da divisão do subescapular é tecnicamente exigente e existe o risco de danos nos nervos devido às forças de tração dos afastadores durante a cirurgia aberta. 21

A integridade estrutural e funcional do subescapular após o procedimento de Latarjet a longo prazo é atualmente desconhecida. 22 23 Em acompanhamento médio de 8 anos após o procedimento de Latarjet, Azizi et al. 22 encontraram ligeira redução da força de abdução, mas não encontraram redução da força de rotação medial. Ademais, em estudos de ultrassonográficos e de ressonância magnética, o tendão do subescapular permaneceu intacto em circunferência quando comparado às medidas pré-operatórias. Em contrapartida, Ernstbrunner et al. 23 depararam-se com diminuição significativa da rotação medial e lateral ativas e da força quando comparada com o ombro contralateral saudável, embora a influência clínica desses achados ainda não tenha sido definida. Não houve aumento da degeneração gordurosa do músculo subescapular, mas uma hipertrofia mínima no lado operado no acompanhamento em longo prazo.

A discussão sobre a integridade do músculo subescapular e sua inervação no procedimento de Latarjet é crescente na literatura. Davey et al. 24 analisaram através de uma revisão sistemática se o manejo do músculo subescapular com divisão ou tenotomia em “L” influenciaria nos resultados clínicos do procedimento de Latarjet aberto por meio de uma abordagem deltopeitoral. A revisão sistemática estabeleceu que a técnica por divisão do subescapular resulta em medidas de resultados funcionais significativamente melhores e taxas significativamente mais baixas de insuficiência do subescapular quando comparada com uma técnica por tenotomia em “L” no acompanhamento de médio prazo. 24 Outrossim, Raiss et al. 21 descreveram o procedimento conhecido como “ *flipped* Latarjet”, isto é, a transferência do coracoide para a glenoide deficiente sem dividir o músculo subescapular, mantendo os benefícios de um efeito de tipoia do tendão conjunto. Embora não existam dados de longo prazo disponíveis para provar qualquer superioridade do método em comparação ao procedimento tradicional aberto ou artroscópico de Latarjet, esta técnica seria uma alternativa para evitar lesões neuromusculares ao subescapular.

As limitações deste estudo são: tamanho da amostra dada pela dificuldade inerente aos estudos cadavéricos, as variações da anatomia do plexo braquial e a perda da tensão das estruturas neurológicas, tendo em vista que foram utilizados cadáveres frescos sem técnicas de fixação da cintura escapular e do úmero. Ademais, a média da idade dos cadáveres utilizados está longe da idade-alvo em que a técnica de Latarjet é utilizada; portanto, aspectos como a qualidade e o volume do músculo poderiam ser modificados.

Por fim, este é um trabalho-piloto cujo intuito principal é verificar a média das distâncias neurológicas, e todas as medidas realizadas pelo mesmo observador foram feitas mais de uma vez para garantir a confiabilidade dos dados. Um estudo complementar deste trabalho está em desenvolvimento, com uma maior amostra e com outro tipo de delineamento para avaliar a confiabilidade interobservador das medições através do coeficiente Kappa.

## Conclusão

A via deltopeitoral é considerada segura; porém, é preciso atenção e cautela durante o *split* do músculo subescapular e na fixação do enxerto do coracoide no procedimento de Latarjet, devido à curta distância até os nervos adjacentes. Tais precauções podem evitar maiores complicações pós-operatórias.
